# Changes in stress-related outcomes among graduate students following the Mindfulness Ambassador Program: A pilot study

**DOI:** 10.1371/journal.pone.0313499

**Published:** 2025-01-16

**Authors:** Varsha Vasudevan, Benjamin Tran, Shauna M. Burke, Patricia Tucker, Jennifer D. Irwin

**Affiliations:** 1 Health and Rehabilitation Sciences Program, Arthur and Sonia Labatt Health Sciences Building, Western University, London, Ontario, Canada; 2 School of Health Studies, Arthur and Sonia Labatt Health Sciences Building, Western University, London, Ontario, Canada; 3 Children’s Health Research Institute, Lawson Health Research Institute, London, Ontario, Canada; 4 School of Occupational Therapy, Elborn College, Western University, London, Ontario, Canada; The Open University of Israel, ISRAEL

## Abstract

**Objectives:**

Graduate students face numerous demands, high stress levels, and associated challenges to intra- and inter-personal relationships. Mindfulness may help to ease such challenging experiences. The Mindfulness Ambassador Program (MAP) is a promising group-based program that has not yet been studied among graduate students. The primary objectives of this study were to: (1) explore graduate students’ perceptions of stress, and their relationships with themselves and meaningful others; (2) explore graduate students’ perspectives of and satisfaction with the MAP; and (3) investigate if participation in the MAP elicited changes in graduate students’ perceived levels of stress, self-awareness, interpersonal skills, and/or social connectedness.

**Methods:**

In this one-group, pre/post mixed-methods pilot study, nine participants completed pre-post questionnaires and participated in a semi-structured interview post-intervention. Data were analyzed using descriptive statistics, thematic analysis, and paired *t*-tests.

**Results:**

Pre-intervention, qualitative themes included participants experiencing moderate-to-high stress levels, intrapersonal conflict, interpersonal relationship challenges, and seeing oneself as a work in progress. Post-intervention themes included better stress management, increased consideration for oneself and others, feelings of connection with others, and overall satisfaction with the MAP. Statistically significant improvements were found from pre- to post-intervention in mean score differences for perceived stress (*p* = .043), private self-awareness (*p* = .006), awareness of immediate surroundings (*p* = .044), and social connectedness (*p* = .006).

**Conclusions:**

Participants reported several benefits from their positive experience participating in the MAP. These findings may be used to inform future mindfulness-based programming for graduate students.

## Introduction

Graduate students face numerous demands that include, but are not limited to, engaging in research, teaching assistantships, coursework, and navigating professional relationships and power dynamics [1]. Compared to those encountered during their undergraduate studies, graduate students are typically faced with greater responsibilities [2]. They are also, on average, older than most undergraduate students, and because their degree-related responsibilities tend to span several years, graduate students often undertake their academic responsibilities while shouldering additional financial, time, and personal commitments [2, 3].

### Stress among graduate students

Studies focused on the mental health and wellbeing of graduate students in North America have increased over the past decade [4–6]. Perhaps unsurprisingly, stress is a pervasive element of these graduate students’ experiences [1, 5–8]. For instance, in a 2022 study of undergraduate and graduate students in Canada, 47% of participants reported feeling a moderate level of stress, and about 37% reported experiencing high stress levels [9]. Similarly, in a 2023 study focused only on graduate and professional students in the United States, 47% of respondents felt a moderate level of stress [10]. Further, in a recent Canadian study of psychology graduate students (*n* = 62), 33.87% of participants reported problematic stress levels [11]. The COVID-19 pandemic has exacerbated graduate students’ stress, and their ability to manage stress has decreased when compared to pre-pandemic times (e.g., [12, 13]). Excessive stress can contribute to physiological and psychological challenges (such as impaired memory and risk of depressive disorder) across the lifespan [14] and there is an association between stress and diminished academic performance among medical students [15, 16].

Graduate student stress can have negative impacts to both students’ academic progress [10] and to the post-secondary institution, as graduate students contribute to the quality and quantity of teaching and research at their institution [17]. Further, graduate students may leave their degree training, or research altogether due to adverse experiences [17]. As such, it would be advantageous for institutions to further invest in and support graduate students’ wellbeing [1, 18]. One such investment might be mindfulness training.

### Benefits of mindfulness among graduate students

Mindfulness is “the awareness that emerges through paying attention on purpose, in the present moment, and nonjudgmentally to the unfolding of experience moment by moment” [15 p.145]. Several benefits to practising mindfulness have been identified, including improved well-being and decreased stress among adults [19], improved immune function among adults [20], regulation of emotions among novice meditators [21, 22], and improved relationship satisfaction among couples [23].

Mindfulness-based stress reduction (MBSR) is a widely researched group-based program focused on encouraging mindfulness as a means of reducing stress and managing emotions [24–27]. MBSR has been associated with lower levels of perceived stress among graduate students [28–30], healthcare professionals [31], and premedical and medical students [32, 33]. For instance, in a study conducted in the United States, MBSR was associated with a reduction in graduate students’ perceived stress levels [30]. A brief MBSR offering also had promising positive impacts on Canadian nursing students’ self-awareness [34].

Self-awareness, an integral component of mindfulness, can be described as knowledge about oneself [35]. Graduate students might struggle with their self-awareness as they are prone to imposter syndrome [36–38], which has been defined as “the experience of intellectual fraudulence or phoniness among high achievers” [35 p.45]. Individuals with imposter syndrome often place lofty expectations on themselves which can be associated with frustration, low self-confidence, and perfectionism [36, 39]. Perfectionism, in turn, can impact students’ academic success, skew self-awareness [39, 40], and damage interpersonal relationships, further contributing to feelings of stress [41].

Mindfulness might also positively contribute to interpersonal interactions [42]. Interpersonal skills are mechanisms used by an individual to facilitate relationships with others [43]; examples of interpersonal skills include effective communication and empathy [44], which are relevant to graduate students’ day-to-day responsibilities as they correspond with their supervisors, peers, and undergraduate students. To enhance these skills, Cohen and Miller [28] found that an interpersonal-focused mindfulness training program was associated with significant increases in the social connectedness and emotional intelligence scores of graduate students (*n =* 21) in the United States. The COVID-19 pandemic has contributed to many university students experiencing feelings of loneliness and disconnection from their peers and professors [45].

The Mindfulness Ambassador Program (MAP; [46]) is an evidence- and group-based program designed to enable participants to become familiar with the principles of mindful living, such that they can promote these principles within their own social circles [47]. The MAP is a 12-session program that can be offered over 6 (x 1.5 hours) or 12 (x 1 hour) weeks and covers the following topics: mindfulness basics; paying attention; discovering inside; connecting authentically; practicing gratitude; mind-body connection; emotional intelligence; noticing emotional triggers; exploring open-mindedness; handling conflict skillfully; nurturing compassion; and being the change [47].

Previous research using the MAP is limited but impressive in terms of its impact on individuals with demanding roles. For instance, using a case-study approach, Smith-Carrier and colleagues [47] reported that their sample of secondary school teachers and students in Canada (*n* = 132) who used the MAP approach experienced improvements in their social awareness and relationship building. Additionally, during focus groups, the teachers shared that they gained stress management tools, and students experienced stress reduction through learning relaxation skills [47]. Roach and colleagues [48] studied the impact of the MAP approach among participants of a parent support program in the United States (*n* = 15), where participants reported significantly lower levels of parental anxiety and depression. More recently, healthcare workers reported significant increases in their resilience levels after participating in an online offering of the MAP [49]. A noticeable gap in the literature pertains to the use of the MAP among graduate students.

While it has been well-established that graduate students benefit from mindfulness-based interventions [28–30, 33], a noticeable gap in the literature pertains to the use of the MAP among graduate students. The MAP is comprised of many similar elements to the much more robustly researched MBSR (e.g., both are group-based programs of similar length, where participants meet weekly and have daily homework practices; both programs cover various mindfulness practices such as body scans and sitting meditations; [24, 26, 27, 47]). Despite these similarities, the MAP is a distinct and separate program from MBSR, and the MAP might be especially appropriate for the busy schedules of graduate students [1, 50] due to its flexible delivery model and relatively low participant burden outside of the formal session offerings (e.g., MBSR can include 45-minutes of homework daily; [26]). Additionally, a unique and potentially inviting element of the MAP is its focus on empowering participants to play an active role in conveying the principles of mindful living within their communities, hence the term, Mindfulness Ambassador Program [46].

The research on the MAP is in its infancy yet is promising; the previously documented outcomes of the MAP in the literature align with the outcomes of interest in the present study (i.e., graduate students’ experiences of stress, their relationships with themselves and meaningful others, as well as the impacts on their stress, self-awareness, and interpersonal skills [47–49, 51, 52]). Therefore, the MAP was deemed a suitable choice as the intervention for this study and for novel application among graduate students. The primary objectives of this study were to: (1) explore graduate students’ perceptions of stress, and their relationships with themselves and meaningful others; (2) explore graduate students’ perspectives of and satisfaction with the MAP; and (3) investigate if participation in the MAP elicited changes in graduate students’ self-reported levels of stress, self-awareness, interpersonal skills, and/or social connectedness.

## Methods

### Participants

Although 15 spots were reserved for students who expressed an interest in participating, 10 volunteers completed the baseline questionnaire prior to participating in the intervention. However, only nine volunteers attended the required number of group sessions (four of the six sessions) and completed the post-intervention assessments (semi-structured interviews and post-intervention questionnaire). We retained the baseline open-ended responses data from the one volunteer who was lost to follow-up; however, we excluded the baseline quantitative data of that volunteer given the analysis method of this study (see “Quantitative Analysis”).

Several volunteers (*n* = 7) reported scheduling conflicts and were no longer able to commit once the MAP had started. The mean age of participants at baseline was 30.5 years, and the majority had identified as white (*n* = 7), and as a woman (*n* = 6). Most were enrolled in a Doctoral program (*n* = 6). Most participants stated that they worked part-time (*n* = 6). Participants were graduate students from a variety of faculties inclusive of art and humanities (*n* = 1), engineering (*n* = 1), health sciences (*n* = 2), information and media studies (*n* = 1), business (*n* = 1), medicine and dentistry (*n* = 1), and social science (*n* = 3).

To be eligible for this pilot study, participants had to be: (1) enrolled as a full-time graduate student at the host institution; (2) able to read, write, and speak in English; and (3) able to reliably access an internet-connected device. Participants were excluded if they were participating in any other mindfulness-based interventions. Using an a priori power analysis, a sample size of 12 with a large effect size (*d* = 0.8) and a significance level of .05 was expected to achieve an acceptable power of 0.83. This sample size is consistent with the MAP guidelines, as the maximum recommended number of participants in a MAP offering is 15. Upon receiving ethical approval from the host university (Health Sciences Research Ethics Board #121112), recruitment began. Convenience sampling was used via a mass e-mail sent to all graduate students at the host institution, host institution-affiliated social media postings on accounts relevant to graduate students, and personal posts on social media by the research team. Recruitment occurred over 8 weeks (August 7, 2022 to September 13, 2022) with the aim of recruiting 15 participants to account for ~ 20% attrition over the six-week study duration. The letter of information was available via a Qualtrics^XM^ link, and after reading the letter of information, participants were able to advance to a second Qualtrics^XM^ link containing the eligibility questions and option to provide informed consent. If a participant passed the eligibility questions, they were able to select either “I consent to begin the study” or “I do not consent, I do not wish to participate.”

### Procedure

#### Baseline assessments

Once eligibility was confirmed and informed consent was received, participants completed a baseline questionnaire via Qualtrics^XM^. The questionnaire completed at baseline included demographic questions, three validated scales (measuring perceived stress, self-awareness, and social connectedness), six open-ended questions, and a MAP-specific pre-program questionnaire (see “Measures”). At the end of the questionnaire, participants were redirected to a separate survey containing the MAP-specific pre-program questionnaire. Upon completion of the baseline questionnaires, participants received their MAP workbook (detailed below).

#### Study intervention

The MAP was offered over 6 weeks via Zoom during the Fall 2022 university semester (September 13 –October 18) in Ontario, Canada. The decision to host this intervention virtually was suitable for pandemic-times and was anticipated to reduce participant attrition; online programming has been found to be of particular value for graduate students due to increased flexibility, reduced travel time, enhanced comfort, and fewer associated costs [53]. A 6-week program delivery (1.5 hours/week) was chosen to fit into a single school term to avoid potential challenges with students’ schedule changes from one term to the next.

The program was offered by a certified MAP facilitator (BT), who was blinded to the study data until the data analysis phase. At the beginning of the MAP sessions, the facilitator encouraged participants to limit distractions (e.g., phones, school-related materials) to help participants stay focused and present during the session. To protect participants’ privacy, all members were asked to join the weekly Zoom meetings from a private location, and/or use headphones to keep the conversations limited to the program participants. Participants were not required to keep their cameras on but were encouraged to do so, as it helped to maintain a sense of presence and connectedness with the group. The primary focus of the group time each week was to facilitate conversation among the participants, based on their experiences with the previous week’s content and the homework activities–this feature creates opportunities for shared learning among group members, per typical MAP offerings [52, 54]. The group-based component of the MAP is consistent with other widely researched mindfulness programs, such as MBSR [26]. Having a group-based element in mindfulness programs is supported in the literature as beneficial to participants [55, 56], and group-based programming is also particularly helpful for graduate students [57–59]. The facilitator moderated the group conversations and reviewed the lesson from the previous week and offered didactic learning opportunities, such as providing definitions of key terms presented in the workbook, to ensure that all participants understood the concepts and knew how to properly engage in the practices. A mindfulness practice was also incorporated during each session, and participants were encouraged to find a comfortable surface on which to sit or lay during this portion. In consultation with the facilitator, participants were permitted to miss up to two of the weekly Zoom sessions, provided they completed the required workbook activities for that week. This provision was implemented to afford more flexibility to the participants. In addition to attending the weekly group sessions, participants were asked to complete a daily breathing practice and a series of homework practice/activities each week; however, the homework activities were not tracked for completion. Practices/activities included guided meditations that could be accessed via a SoundCloud link, assigned readings, and reflective questions included in the MAP workbook. The homework practices/activities in the workbook were designed to help participants build the skills associated with each session (e.g., breathing mindfully, listening deeply, and practicing compassion) and took between 15 and 30 minutes to complete each week.

#### Immediate post-intervention assessments

Once the last session of the MAP concluded, participants were sent an email inviting them to schedule a semi-structured interview with the lead researcher (VV) to discuss the influence of the program on their levels of stress, and their relationships with themselves and meaningful others. The email also contained a link to a Qualtrics^XM^ questionnaire to assess participants’ post-intervention levels of stress, self-awareness, interpersonal skills, and social connectedness, using the same tools used during the baseline questionnaire (outlined below). A link to the MAP-specific post-program questionnaire was also included and participants had one week to complete both online questionnaires.

#### Measures

Using a mixed methods approach for pilot studies is encouraged to gain a more robust understanding of participants’ experiences of an intervention [60–62] and aligns with the post-positivist paradigm from which this study was conducted [63, 64]. A post-positivist approach acknowledges that knowledge is relative rather than absolute and supports the usage of both qualitative and quantitative approaches to better understand how the world operates [63]. Using multiple methods is supported by acknowledging that each method has its own limitations [63]. Therefore, the mixed method approach used in the present study offered an opportunity to collect data that were complementary and holistic.

#### Demographic questions

Demographic questions included participants’ age, gender, ethnicity, employment status, year of enrolment, and faculty of registration. Demographic data were collected at baseline.

#### Open-ended questions

As part of the baseline questionnaire, participants responded to the following six open-ended questions: (1) Please describe your relationship with yourself; (2) Please describe your relationship with your meaningful others; (3) Please describe your current stress levels; (4) Self-awareness can be described as “knowledge about the self.” Please describe the extent to which you perceive yourself to be self-aware; (5) Interpersonal skills can be thought of as mechanisms used by an individual to maintain their relationships. Please describe the level of satisfaction you currently experience with regard to your interpersonal skills; and (6) What do you hope to gain from your participation in this program?. These data were collected to glean participant perspectives before participating in the MAP. Post-intervention, participants were asked to provide responses to the following three open-ended questions (in addition to other tools detailed below) about their experience via the MAP-specific post-program questionnaire: (1) What were the most valuable aspects of this learning experience for you?; (2) How could this learning experience be improved?; and (3) Do you have additional comments or reflections?.

#### Semi-structured interviews

Semi-structured interviews were conducted post-intervention using a nine-question guide developed by the research team. The interviews generated qualitative data as part of the mixed methods approach, which afforded the research team a richer understanding of participants’ experiences during the MAP, representing was an important contribution of this pilot study. In fact, for this reason, the use of mixed methods for pilot studies has been recommended in the past [60–62].

Participants were asked to reflect on changes to their levels of stress, self-awareness, and interpersonal skills post-MAP, any other changes or impacts they noticed since participating in the program, and the extent to which they thought the MAP was practical for graduate students. The added effect of the interviews was that we learned program-specific suggestions for improvement which would not have been gathered using our other data sources. The interviews were conducted virtually over Zoom and lasted between 22 and 60 minutes. Prior to starting the interviews, participants provided verbal consent for the interview to be recorded for transcription purposes. During the interviews, to support data credibility, member checking of participants’ responses was conducted by the interviewer (VV) to verify that what was being understood by the researcher was what the participant intended. Automatic audio transcription, a feature on Zoom, was utilized. Upon completion of the ninth interview, it was clear data saturation had been reached [65]. At baseline and post-intervention, the following four tools listed below were implemented.

### Perceived Stress Scale (PSS)

The PSS measures how an individual appraises different life events as stressful (e.g., how often one has felt nervous and stressed in the past month; [66]). The PSS-10 was chosen for the purpose of this study as it is considered to have the strongest internal validity compared to other versions of the PSS [67] and has been validated specifically for use in the university student population (Cronbach’s α > 0.8; [68]). It includes 10 questions, with five corresponding response options (never, almost never, sometimes, fairly often, and often) which are scored 0 through 4, respectively; questions four, five, seven, and eight are reversed scored [69].

### Situational Self-Awareness Scale (SSAS)

The SSAS measures an individual’s public and private self-awareness levels [70]. It consists of three subscales (public self-awareness, private self-awareness, and self-awareness of surroundings) and each of the subscales includes three items, for a total of nine items [70]. The three subscales are individually scored by summing the responses to the three items pertaining to each subscale. There are seven response options for each of the nine questions, with options ranging from 1 (strongly disagree) and 7 (strongly agree). An example item is “right now, I am conscious of my inner feelings” [53, p.369]. This tool has been deemed psychometrically sound in undergraduate-level university students (α = 0.82 for public self-awareness, α = 0.70 for private self-awareness, and α = 0.72 for self-awareness of immediate surroundings; [70]).

### The Social Connectedness Scale–Revised (SCS-R)

The SCS-R [71] was used to gauge the interpersonal closeness of participants with their social connections, where social connectedness is considered a form of a “cognitive structure representing regularities in patterns of interpersonal relatedness” [72]. The SCS-R consists of 20 items, and each question has six corresponding response options ranging from 1 (strongly disagree) to 6 strongly agree [71]. The scale measures the extent to which one perceives themselves as feeling connected to others (e.g., “I am able to relate to my peers”; [54, p.312]). A score is generated by reverse scoring certain items and then summing all 20 items. The SCS-R has been previously validated for use with undergraduate university students (Cronbach’s α = 0.92).

### MAP-specific participant questionnaires

The MAP protocol includes participants completing a pre- and post-program MAP-specific questionnaire. These questionnaires are not validated, and no formal scoring protocol exists, as they were created for internal use and evaluation by the creators of the MAP. Data from the MAP-specific questionnaires were included in this study for three reasons. First, they were designed to assess specific constructs of the MAP and the research team deemed them to hold face validity. Second, because this was a pilot study, the research team used the advice of Aschbrenner and colleagues [60] to err on the side of including more rather than less data to understand the experience of the intervention. Third, the foci of the MAP-specific items aligned with outcomes of interest for this study–self-awareness and interpersonal skills—thus, the research team deemed them relevant to include, as they could be helpful for providing a more robust understanding of participants’ experiences with the program. Findings from the MAP-specific questionnaires were used to supplement the other data collected as part of this study. The pre-program questionnaire consisted of 12 items prompting self-reflection, with five response options ranging from “Never” to “Almost Always.” The post-program questionnaire included three sections; the first section included the same self-reflection-focused 12 items used in the pre-program questionnaire; these questions specifically addressed self-awareness and interpersonal skills outcomes. Summing the responses pertaining to each outcome to obtain a score for both time points was deemed suitable. The second section of the post-program questionnaire consisted of five statements asking participants to reflect on their satisfaction with the MAP on a five-point scale (ranging from never to almost always). The third section included six questions, which were a mix of yes or no, descriptive, and open-ended questions that asked participants about the extent to which they shared their MAP-related learnings with others, and their reflections on the program’s value and recommendations for potential improvements.

### Data analyses

#### Qualitative analysis

To explore participants’ perspectives of stress, their relationships with themselves and others, as well as their experience with the MAP, data from the open-ended questionnaire responses and transcripts from the semi-structured interviews were analyzed using thematic analysis [73]. Conducting qualitative research from a post-positivist paradigm involves the researcher taking an objective stance to minimize potential bias [63, 64, 74]. For the open-ended questions from the baseline questionnaire, the lead researcher (VV) and a research assistant (JW) independently reviewed and coded the responses to minimize bias and support data confirmability [63, 64, 74]. Data confirmability—an element of post-positivism—refers to presenting the findings such that they represent the situation and not the beliefs or biases of the researcher [65]. Upon completion of the coding, the researchers convened to determine the final themes along with their corroborative quotations [74]. Based on the recommendations of Floyd and colleagues [75], for the semi-structured interviews, only audio recordings were used during data analysis to avoid challenges such as the possibility of increasing implicit bias by analyzers based on participant appearances. The same thematic analysis process used to code the open-ended responses was conducted to analyze the interview data; however, peer debriefing [74] also occurred between the interviewer and the research assistant (RK) while analyzing the interview transcripts.

The analyses presented in the following section (see “Results”) are consistent with the thematic analysis procedure employed to analyze our data, where we tell the research story through the themes presented in the next section, then, situate our findings and continue our analytic narrative in the discussion [73]. Thus, the “Results” section prioritizes quotations to highlight the experiences of the participants, rather than researchers’ interpretations. While the selected quotations may be applicable to multiple themes, each has been shared only once in the theme to which it most aligns. Additionally, illustrative quotations, rather than the frequency of instances related to a theme, are used to highlight the prominence of each theme, per Braun and Clarke [73]. Although the interview recordings were transcribed verbatim, the quotations were edited using a denaturalized approach to omit utterances and duplicate words to improve the readability and clarity for readers [76]. Please note that in the following section (see “Results”) we were intentional in using gender neutral pronouns when reporting participant quotes due to our small sample size and our commitment to de-identifying participant quotes to the best of our abilities. We believe the sentiments of the qualitative data were rich and provided strong insights to the MAP participants’ experiences; therefore, de-identifying the gender of the participant would not interfere with the presentation of the quote. We reported detailed demographic details in support of the study’s potential transferability.

#### Quantitative analysis

Quantitative data analysis, using IBM SPSS Statistics (version 28), involved computing measures of central tendency and dispersion (descriptive statistics), the scoring of the validated scales, as well as paired *t*-tests. Descriptive statistics were used to analyze the program satisfaction tool from the MAP post-program questionnaire. Paired *t*-tests were used to analyze changes in the PSS, the SSAS, the SCS-R, and the 12-item tool from the MAP pre- and post-program questionnaires.

## Results

### Pre-intervention open-ended survey response findings

Four major themes (and three subthemes) were identified at baseline: (1) moderate to high stress levels juggling competing responsibilities; (2) intrapersonal conflict (subtheme: conflict between awareness and action); (3) difficulty connecting with meaningful others; and (4) being a work in progress (subthemes: (a) stress management from the MAP; and (b) intrapersonal development from the MAP).

### Moderate to high stress levels juggling competing responsibilities

Participants’ written responses to the open-ended survey questions described moderate to high stress levels about their competing academic and personal responsibilities. When asked about their current stress level, one participant wrote, “high stress. Life as a grad student, trying to be a loving spouse, trying to be a good parent, trying to not disappoint my advisor’s seemingly unreasonable expectations. It’s like they design this program to destroy your sanity? **☺** Joking, not joking” (P10). The assortment of responsibilities that participants discussed often came with a sense of urgency, as demonstrated by another participant who said, “I am working full time until the end of this week, I have comprehensive exams on the [date] and am moving to [city] on the [date]. Plus, I also have to somehow put together a [scholarship] application and do a class remotely” (P2).

### Intrapersonal conflict

Participants described conflicting views about themselves, with a level of tension regarding their intrapersonal relationship. For example, one participant explained, “I have a good relationship [with myself] but can also be my harshest critique. A lot of my self-validation comes from doing well academically and having success with my research, so when I encounter challenges that can be tough” (P1). Another participant described conflicting views about themselves by writing:

I regularly analyze different aspects of myself (facial expressions, body language, communication skills, actions that were or were not taken, etc.) but my perception is typically tainted with a negative lens, quite a bit of fear and anxiety, and so I don’t think my perceptions are always accurate. (P6)

### Sub-theme: Conflict between intrapersonal awareness and action

Despite feeling varying levels of internal conflict regarding their self-awareness, several participants described having an awareness of their needs; however, they also reportedly struggled to attend to those needs, often due to competing demands on their time. For example, one participant wrote:

I have a tendency to react [in] certain ways in certain circumstances (for example, when I’m busy working on something, I often forget to eat, and then I feel irritable), but that doesn’t always help me avoid those reactions in the moment (e.g., I forget to eat, and the logical part of my brain is telling me, “I’m feeling cranky because I haven’t eaten anything lately” but I still struggle to actually do anything about it). (P3)

Another participant echoed this by writing, “I feel like I should take more time to reflect, but I feel like I don’t have time to reflect. I don’t have time to figure things out because I have too much stuff to do that needs to get done” (P10).

### Difficulty connecting with meaningful others

Connecting with meaningful others was a challenge reported by most participants and there was a considerable amount of variation in the responses regarding participants’ interpersonal skills. Several specific reasons for this challenge were shared by participants, although the innuendo of isolation seemed common to them all, including: “differing levels of risk-tolerance [due to the pandemic]” (P3); “feel[ing] stressed and overwhelmed with school sometimes which impacts my relationship with others” (P1); and not “feel[ing] comfortable talking about my issues with anyone and feel[ing] like I have too much trauma for anyone to ever be able to relate to it” (P2). Participants also attributed their difficulties in connecting with meaningful others to the aptitude of their interpersonal skills. One participant wrote, “my interpersonal skills are lacking. If I were left to my own devices, I would likely become isolated” (P9). Another participant wrote, “with the pandemic I was working a lot at home independently, so I think I lost some of those interpersonal skills because I wasn’t having to interact with people on a regular basis” (P1).

Some participants stated they had meaningful relationships and felt strong connections with certain individuals. Among those who reported having positive relationships with meaningful others, all but one participant said that the positive relationship to which they were referring was with their partner or spouse. One participant explained, “[regarding] my significant other, our relationship is healthy and growing but relatively new and so we are still figuring things out” (P6). A second participant echoed the quality of their closest relationships saying, “I have a loving partner that I adore, a strong group of friends, and some family relationships” (P4).

### Being a work in progress

Many participants considered themselves to be a work in progress. Although self-criticism was evident throughout their responses, so too was an acknowledgement of participants’ progress in their personal journeys. For instance, one participant wrote:

I find myself frequently engaging in a lot of negative self-talk around my self-worth, communication skills, ability to take on leadership roles, lack of energy towards/engagement in exercise, academic coursework, and extracurricular activities, and ability to maintain professional/interpersonal relationships … Although I still struggle with these things, I have come quite far over the past 1.5–2 years. I am better at (but still working on) setting boundaries and taking rest when I feel tired. (P6)

Another participant described the notion of being a work in progress by writing, “I’m trying to figure myself out. How to be more patient and caring (and not frustrated or upset) towards those I love and how to be more focused and diligent in the work I do” (P10).

### Sub-theme: Stress management from the MAP

Participants hoped to learn how to better manage their stress by participating in the MAP. One participant wrote, “ways to manage stress or better understanding my stress would be what I hope to gain most [from the MAP]” (P7). Another participant wrote, “I’d like to develop better strategies for helping me manage stress, especially at times of the year when I have a lot of competing deadlines or demands on my time” (P3).

### Sub-theme: Intrapersonal development from the MAP

Participants indicated wanting to develop their intrapersonal skills, specifically, participants described wanting more self-awareness and improvements to their mindfulness skills. For example, one participant wrote, “I am hoping to learn how to take time for myself and deal with the stress I may encounter in grad school. Specifically, strategies I can use to minimize it and improve relationships with those around me” (P1). Another participant wrote that they hoped to gain “more self-awareness and self-confidence. … I hope to build the habit of setting aside some time each day/a couple times a week to check in with myself and become more attuned to what I am feeling or need” (P6).

### Post-intervention qualitative and quantitative findings

Five themes and four sub-themes emerged from the data pertaining to participants’ experiences in the MAP: (1) improved stress management; (2) increased consideration for oneself (subtheme: increased awareness of the self); (3) increased consideration for others (subtheme: deep listening); (4) feelings of connection; and (5) logistics (subthemes: (a) virtual format; and (b) program timing and length).

### Improved stress management

The majority of participants reported their engagement with the MAP brought forth an increased awareness of the stress and stressors they were experiencing, and this awareness helped some to better manage stressful experiences. For example, one participant conveyed, “I’m kind of realizing ‘okay, I’m actually being stressed right now’ … I am objectifying the stress as a thing that I can be aware of rather than just experiencing it” (P8). Specific to stress management, some participants described that, since participating in the MAP, they felt more in control when experiencing stress and did not feel as overwhelmed by stressful times. A participant described a major stressor in their life and how the MAP was able to provide some support. They said:

my partner had a medical emergency 2 weeks ago … he had an emergency surgery. So that really increased my stress level … but I found myself less reactive during this difficult time than I would have been in the past. So, I think that the MAP actually helped me deal with this very stressful emergency … usually [pre-MAP] I would be freaking out and very anxious. I was still anxious, but I feel like it would have been worse without the MAP, and I didn’t skip any sessions, even on the day of my partner’s surgery I attended because I actually wanted to attend MAP. It [helped] me go through this. (P5)

### Increased consideration for oneself

Participants described being more considerate of themselves post-MAP, offering examples such as taking time for themselves, being kinder to themselves, and changing academic outlooks. One participant described a situation when their focus was derailed:

often, what I would do [pre-MAP] is just sort of go into this kind of spiral of not getting anything done all day, and instead this time I kind of noticed myself doing this and … took half an hour to step away from my computer and … just do things that I find a little bit more relaxing and I was sort of able to get back to working … I guess that was the difference … recognizing in the moment that I needed to do something that maybe didn’t look productive in order to be productive again. (P3)

Several participants recognized that they wanted to be kinder to themselves, especially in academic contexts. For instance, one participant conveyed:

the biggest takeaway that I took from the program was this idea of being kind to myself. Beforehand, I felt I had to have really exacting standards. Because, you know, I wanted to be a good [spouse]. I wanted to be a good [parent]. I was in a really difficult, world-class program, I wanted to deserve to be there … make my advisor and everyone feel like they made the right decision in terms of choosing me and so on … if I couldn’t meet exact standards then there was a lot of [self-talk of] like “you suck.” … [the MAP], you know it’s sparked that idea that it makes sense to be kind to myself. (P10)

### Sub-theme: Increased awareness of the self

Following participation in the MAP, a few participants discussed feeling more considerate of themselves as they better attended to their needs because of their increased awareness. They described being more adept at recognizing the signs when something required attention, as demonstrated by the following quotation:

[post-MAP] I’ve become a little bit more aware of [my anxiety] and just a little bit more responsive to my own needs. If I need to take a minute and do some self-care … I’ll do that now. … I feel like for the most part it has *slowly* gotten better to the point where I can recognize what my body is telling me … and what I need. (P6)

Another individual recounted how, since being in the MAP, they developed greater self-awareness and were able to identify signs that something required their attention. They said:

the biggest [take]away from [the MAP] was just taking time to recognize what the signs are, in myself … I would get headaches and so I think I’ve started to recognize it’s when I’m really tired of staring at a screen. And so just recognizing, taking those breaks has been important … the program enforced [me] to think about those [signs] a lot more than perhaps I was before … those signs haven’t changed, it’s just now that I’m more aware of, “oh, if I like have a bad headache, what is that from?” … just kind of being more aware of sort of what my body needs … has been a big thing. (P1)

### Increased consideration for others

Participants expressed increased consideration of others after MAP participation, which seemed to facilitate a change in some of their perspectives. For example, one participant said:

my stressors in life are different than some of my friends who aren’t in school anymore and … they’re equally going through stressful times, it’s just different for them than what it is for me. … that perspective of taking a moment to realize … you can’t really see everything that’s going on with them but just recognizing that they … could still be experiencing some challenges or difficulties, they’re just different than yours. (P1)

One participant described improvements in their interpersonal communication since their MAP participation. They said: “[since attending the MAP] I’m … responding to text messages, more often than before, actually making an effort to acknowledge people even if I don’t have time to see them, at least keeping that line of communication open … it’s a work in progress” (P9).

### Sub-theme: Deep listening

Deep listening was a communication strategy from the MAP described as paying attention to a speaker without judgement or expectation and with a quiet mind, to which participants were particularly receptive. Some participants realized they were often formulating their responses while listening to others instead of listening deeply. One participant expressed their intention to improve by saying, “I just really wanted to make sure that I was listening to them, and not just thinking about how to solve their problems, [which distracts from] my listening” (P2). Another participant shared a reflection they had regarding deep listening saying, “it’s okay, if … I don’t say the perfect thing but as long as someone knows I’m there” (P7).

### Feelings of connection

Participants valued the opportunity to connect with the other graduate students participating in the MAP. The importance of partaking in the MAP as a group was underscored by participants, who described being engaged in meaningful conversations despite being essentially strangers. The conversations during the sessions were deemed relatable, often from an academic perspective, which seemingly contributed to feeling understood and helped lessen feelings of isolation and/or loneliness. For instance, one participant commented, “[sharing with other participants] was very helpful to make me feel connected to my fellow grad students, because a lot of them share my struggles in slightly different ways and being able to listen to their stories makes me feel less alone” (P5). Conversations during the MAP sessions seemed to help participants reframe feeling alone in their struggles to feeling more connected to graduate students as a whole. Participants discussed feelings of academic connection too, stating they were often one of the few individuals in their social circles still in school. Another participant’s comment resonated with this notion. They said:

most of my friends aren’t graduate students … so, their stresses are so different from my stresses that truthfully, they just don’t get it. I just went through my comprehensive exams, and they [friends] just didn’t get why or what actually writing a comp[rehensive] exam is like … there [were] points [during the MAP] where … there was a general understanding in the [virtual] room where everybody’s just been through the sort of baseline [graduate student] experience and so they all have that kinship. (P2)

### Logistics

The logistics theme includes two attributes of the MAP: (1) the delivery of the program using a virtual format; and (2) the program timing and length.

### Sub-theme: Virtual format

Offering the MAP virtually (using Zoom) was very well-received by participants. Participants described that one of the greatest benefits of offering the program virtually was that it afforded them the ability to be more vulnerable and engage more authentically with the MAP and each other. The virtual format allowed the participants to be in the comfort of their own environment when discussing topics that were personal or sensitive. Additionally, although participants were not required to keep their cameras on during the MAP sessions, most did keep their cameras on for the whole duration of each session with the exception of the mindfulness practice portions. Participants appreciated the ability to turn on/off their camera, especially during the mindfulness practices. For example, one participant said “I would have felt like I couldn’t … let go of control [of my emotions] in the same way. … I would have been conscious of there being people around me watching me and stuff like that” (P3). Another participant shared similar sentiments as included in the previous quote. They said:

[the virtual environment] create[d] … distance that makes me feel comfortable. … I know that some people prefer in-person group meetings, that makes them feel more connected to the fellow humans, but for me, that makes me very uncomfortable to sit in the same room face-to-face with strangers … the Zoom format really worked for me. (P5)

However, one participant did mention that they would have felt more secure during the sessions if everyone was in the same room. They explained that:

sometimes people were accessing [the MAP] from a public space and so they might have had headphones in … it just doesn’t feel as safe and secure as it would have been if we’re all in a room together and there’s no one else there. (P1)

Another benefit to the program being offered via a virtual format was that participants found it more accessible; they explained that joining the MAP sessions virtually reduced travel time and allowed them to fit this program into their busy schedules. One participant, who was living in a different city than the host institution said, “I couldn’t have done this program if it was in person right, so I think [the virtual format] still gives me more opportunities” (P2). Another person appreciated the accessibility of the virtual format and said:

I usually pick Zoom [when there is the option to so] just, you know, in case something comes up, and I’m not … able to get to campus that day … with some of my anxiousness, it’s easier to kind of hide on a screen than if you were in person. (P7)

### Sub-theme: Program timing and length

Regarding the program timing and length, participants’ suggestions included meeting over 12 weeks instead of 6, changing the time in which the sessions were offered, and providing more opportunities to attend or to miss sessions. One participant wrote, “I wonder if having the sessions separate may have been useful to spread out what we’re learning (so meeting 12 times instead of [including] two [of the MAP] sessions in [each of the] 6 meetings)” (K29HB81).

Participants mentioned they would have appreciated more flexible time options besides the 11 a.m. Eastern Standard Time session each week. Many participants mentioned the time of day was inconvenient, stating they would be productive in the morning and felt like they had to stop their flow of work to attend to the MAP session. One participant said:

I just wasn’t as focused as I feel I would have been if maybe it was later in the day or earlier and I don’t know, my emails [were] still going off … it’s a popular time for people to be sending emails and so I’m just sitting here like “Oh, I need to respond to these” but I need to focus on [the MAP session] at the same time” (P1).

Some participants mentioned they would have appreciated more options from which to choose each week, as reflected in the following quotation, “if they had more frequent sessions or … more flexible scheduling times that would make it, I guess even better for graduate students because they are typically busy” (P8).

### Quantitative findings

Participants’ satisfaction with the program is presented in Table 1. Across the five statements related to satisfaction, the average response was approximately four out of five. The difference between the pre- and post-intervention scores for stress, self-awareness, interpersonal skills, and social connectedness are presented in Table 2. From pre- to post-intervention, there was a statistically significant decrease in participants’ perceived stress (*p* = .043). Participants’ private self-awareness increased after participating in the program (*p* = .006). The self-awareness of immediate surroundings subscale showed a significant positive change from baseline to post-intervention (*p* = .044). Participants experienced significant increases in social connectedness from pre- to post-program (*p* = .006). There was a significant increase in the self-awareness-coded items from the MAP-specific pre-program questionnaire (*p* = .006).

**Table 1:** Program satisfaction results from the MAP-specific post-program questionnaire.

Question	*N*	Mean	Minimum	Maximum	*SD*
Overall, I was satisfied with the learning experience	9	4.33	3	5	.87
The content of the learning experience was appropriate for my needs.	9	4.11	3	5	.93
I gained a solid understanding of the topics covered in this learning experience.	9	4.44	3	5	.73
It’s clear how I will apply the knowledge gained from this learning experience.	9	4.11	2	5	1.05
This learning experience made good use of time.	9	4.22	2	5	1.09

Note: Participants selected a score from the following options: never (1), rarely (2), once in a while (3), sometimes (4), and almost always (5); Standard Deviation (*SD*)

**Table 2:** Difference in means between baseline and post-intervention scores for perceived stress, situational self-awareness, social connectedness, and items from the MAP-specific questionnaires.

Paired *t*-test	Baseline Means	Post-Intervention Means	Difference in means (Post-Intervention–Baseline)	95% Confidence Interval	*SD*	*p*-value	Post-hoc power	Effect size (Cohen’s *d*)
PSS	29.222	25.222	-4.000	[-7.843, -.156]	5.000	.043	0.559	-.800
SSAS (Private)	12.222	15.556	3.333	[1.228, 5.438]	2.739	.006	0.891	1.217
SSAS (Public)	14.222	12.111	-2.111	[-5.382, 1.160]	4.256	.175	0.259	-.496
SSAS (Immediate Surroundings)	12.333	15.333	3.000	[.098, 5.902]	3.775	.044	0.554	.795
SCS-R	63.778	80.111	16.333	[6.064, 26.603]	13.360	.006	0.893	1.223
Self-awareness items	30.000	36.000	6.000	[2.274, 9.726]	4.848	.006	0.900	1.238
Interpersonal skills items	11.667	12.444	0.778	[-.843, 2.398]	2.108	.301	0.163	.369

Note: Perceived Stress Scale (PSS); Situational Self-Awareness Scale (SSAS); Social Connectedness Scale-Revised (SCS-R); Standard Deviation (*SD*).

## Discussion

The primary objectives of this study were to: (1) explore graduate students’ perceptions of stress, and their relationships with themselves and meaningful others; (2) explore graduate students’ perspectives of and satisfaction with the MAP; and (3) investigate if participation in the MAP elicited changes in graduate students’ perceived levels of stress, self-awareness, interpersonal skills, and/or social connectedness. Taken together, the findings from this mixed-methods pilot study indicate that participants experienced various stress-related, intrapersonal, and interpersonal benefits from their MAP participation. Furthermore, they found the MAP highly satisfactory. A number of findings warrant discussion.

The significant and meaningful reduction in perceived stress reported by participants after completing the MAP is especially noteworthy given the timing of the program offering (mid-September to late October). Specifically, academic stressors and associated experiences of stress tend to increase from the start to the end of a term [77, 78]. However, participants in the current study described gaining stress-management tools and experiencing a reduction in their stress by the end of the program. This is particularly relevant given Dai and colleagues [79] reported that joining a behaviour change program in concert with other new beginnings is an effective approach for encouraging aspirational behaviour change. Dai and colleagues [79] proposed a ‘fresh start effect’ which describes the galvanizing effect a new temporal landmark (e.g., a new academic semester) has on beginning positive behaviour changes. While there may be momentum for students to begin a positive behaviour change–such as participating in a mindfulness-based program–at the beginning of a semester, having multiple new responsibilities associated with the start of the term (e.g., teaching assistantships, research responsibilities) might also pose challenges to participant engagement and/or attrition. In fact, researchers have underscored that the beginning of an academic year can be a particularly stressful time for students [78, 80]. Navigating transitional stressors (e.g., moving to a new city and other lifestyle changes that coincide with beginning a new degree) can be challenging without established social support networks [80, 81] and data from the present study indicated that the MAP provided participants with tools to mitigate these challenges.

The lack of social support at the outset of a graduate student’s degree can be especially challenging, as low levels of satisfaction with support are associated with negative impacts on perceived stress [82, 83]. Given this research took place during the third academic year of the COVID-19 pandemic, it is likely that all of the participants in the present study began their current graduate degree during the pandemic, as all but one reported being in their first, second, or third year of study. Therefore, the participants in our study may not have had the same opportunities to develop social support systems at the start of their degrees, compared to students who started their degrees before the pandemic [45]. Given the impacts of the ongoing pandemic [84–87], a mindfulness-based intervention aimed at graduate students could be both beneficial and timely. The significant improvement in perceptions of social connectedness among participants in our study might reflect that participation in the MAP could have helped to fill this social support gap. The positive response to the group component of the MAP is consistent with perceptions of group-based learning for graduate students [58, 59]. Given mindfulness-based interventions’ efficacy in reducing stress in post-secondary students [88, 89] and the positive influence mindfulness can have on students’ social interactions [28, 90], offering a mindfulness-based group program at the start of a new academic year with built-in flexibility to accommodate graduate students is something future programmers may wish to consider.

Participants in the current study experienced significant improvements in and provided rich descriptions of positive shifts in their awareness and consideration of themselves. In fact, participants’ descriptions of how participating in the MAP influenced their intrapersonal relationship reflected an overall experience of enhanced self-compassion, per Neff’s [91] characterization of the term. Neff [91] proposed that self-compassion includes self-kindness, common humanity, and mindfulness. Participants’ descriptions of improved self-compassion after completing the MAP are consistent with the literature, as previous studies have shown self-compassion improvements among graduate students and healthcare professionals after participation in mindfulness-based programs [30, 92]. Self-compassion has also been associated with benefits in the general adult (i.e., non-student) population, including lower levels of personal distress [93], enhanced motivation to change self-reported personal weaknesses (e.g., being shy and having social difficulties; [94]), and higher levels of relationship satisfaction of partners and improved relational wellbeing [95]. Self-compassion has also been studied among undergraduate students in the United States who were starting university, and Terry and colleagues [96] found that those higher in self-compassion reported less homesickness and depression, and greater satisfaction in attending university. Further, Swee and colleagues [97] found that undergraduate students in the United States who participated in a brief self-compassionate letter-writing intervention experienced significant reductions in self-criticism. While all of the above-noted benefits of self-compassion appear relevant to graduate students, the decrease in self-criticism found in the study by Swee’s team is especially notable given self-criticism is a part of maladaptive perfectionism [98] and perfectionism can predict feelings of imposter syndrome in graduate students [40]. Given the results from the current study indicate that the MAP was associated with bolstered self-compassion, researchers of future studies might consider including a self-compassion measure to quantitatively capture the MAP’s impact on this construct.

An increase in self-compassion might have contributed to our study participants reporting feelings of connection with others through participating in the MAP. Edwards and colleagues [99] explained that an integral characteristic of practicing self-compassion includes individuals actively recognizing that they are part of a community (as opposed to isolated individuals), which may improve feelings of connection with others. However, Edwards et al. [99] noted that this recognition and appreciation of being part of a larger community is a unique strength of self-compassion and may not be an inherent part of all mindfulness-based programming. In fact, it is important to note that participants underscored that they experienced these connections being made because of the MAP’s group-based programming/discussions. The value of group discussions was also highlighted by graduate students in Willgens and Palombaro’s [92] 6-week mindfulness-based program in the United States. The participants reported benefitting from meeting people outside of their typical academic stream and learning that they were experiencing similar, if not the same, challenges [92]. This notion is consistent with the current study’s participants’ descriptions of recognizing the challenges they face as graduate students being echoed by their peers, which was noted to have helped to mitigate feelings of loneliness. The implications of not feeling alone in one’s struggles are important, as isolation among undergraduate students has been found to predict increases in depression and anxiety levels, worsened mental health, and an increased risk of eating disorders [100]. Although specific to undergraduate students, findings from Richardson et al. [100] are likely relevant for graduate students, as nearly 20% of participants in Ray et al.’s [101] study of graduate and professional health science students in the United States reported being socially isolated. Moreover, graduate students in another study reported not having many opportunities to interact with students outside of their discipline due to their program’s structure [102]. Graduate students tend to have limited formal opportunities to interact with students outside of their discipline [92, 102] which might exacerbate feelings of isolation. The MAP could serve as a forum for graduate students to have time for regular interpersonal interactions, while also benefitting from the other strengths of the MAP (e.g., stress management tools and mindfulness strategies).

Overall, the graduate students in the current study reported being highly satisfied with their MAP experience. In fact, the mean scores for all five questions from the satisfaction-oriented questionnaire never dipped below four out of five, reflecting general satisfaction among participants in terms of MAP’s content, the learning experience, and application. These findings are consistent with MacDougall et al.’s study [52] which found high scores on the Client Satisfaction Questionnaire when assessing the acceptability of the MAP among youth with early psychosis. While, in the current study, ‘satisfaction’ was measured via the non-validated MAP-specific post-program questionnaire, additional content from the qualitative components pointed to some specific outcomes that were particularly satisfying. For instance, pre-intervention, participants expressed an interest in improving their stress management and intrapersonal skills, and both of these skill sets were described as being bolstered post-intervention.

### Limitations and future research

While valuable insights have been identified via this pilot study, it was not without limitations. Although rich and consistent themes emerged from the qualitative data, the small sample size limited the power of the study and, therefore, the quantitative findings. Moreover, given homework completion was not tracked and participants were allowed to miss two sessions, it is difficult to discern the extent to which participants were engaged with the intervention. The sample lacked racial and gender diversity, with the majority of participants being white and identifying as women, which is a consistent trend among mindfulness-based research [103]. Although reaching saturation provided a rich understanding of the experiences of our relatively homogeneous sample, the low sample size and lack of diversity limit the potential transferability of these findings by other researchers to other groups of graduate students whose demographics are more diverse. Lastly, due to the voluntary nature of mindfulness programs and the convenience sampling strategy used, individuals who were interested in mindfulness and/or believed it to be helpful were likely those who chose to participate; this might have contributed to some of the positive feedback [104]. Researchers wishing to explore the MAP’s influence on graduate students should consider purposefully recruiting and studying the impact of the program on a larger and more diverse sample of participants; a more diverse sample would allow researchers to explore gender-based differences of the MAP as well. It would also be valuable to assess differences when offering the program over 6 weeks compared to 12 weeks, while also providing graduate students with the option to choose among multiple session days and times to further enhance the flexibility of the program. Given the target sample size was not reached during recruitment, researchers might consider adding incentives to future program offerings or embedding a mindfulness-based program into a health-focused class to enhance the engagement of graduate students [105]. Additionally, as the present study did not include a control condition, it would be valuable for researchers to study the effects of the MAP compared to a control condition with a powered sample of graduate students. Such a study could support making recommendations about widely implementing the MAP at post-secondary institutions for graduate students.

To the best of the research team’s knowledge, this pilot study was the first to explore the influence of the MAP on a sample of graduate students. The findings from the study indicate that the MAP is a viable approach to help positively influence graduate students’ perceptions of stress, their relationships with themselves and meaningful others, perceived levels of stress, self-awareness, interpersonal skills, and social connectedness, but additional research is warranted. Specifically, these research questions should be explored with a larger and more diverse group of graduate students to further understand the MAP’s potential for graduate students amidst continuously changing landscapes (e.g., during non-pandemic times).
